# Mental health care reform in Belgium: interorganisational networks and integration of care

**Published:** 2012-09-04

**Authors:** Griet De Roeck, Mark Leys

**Affiliations:** VUB, Organisation, Policy and Social Inequalities in Health Care (OPIH), Belgium; VUB, Organisation, Policy and Social Inequalities in Health Care (OPIH), Belgium

**Keywords:** integrated care, mental health care, interorganisational networks, network complexity

## Abstract

**Purpose:**

To make a plan evaluation of projects being part of major mental health care reform process in Belgium. It emphasizes on the logic, underlying considerations and choices for network configurations developed to provide adequate mental health care.

**Background:**

In 2011 a large-scale reform process in mental health care was launched in Belgium. The reform aims at deinstitutionalization and integration of care. Within the constraints of a general policy programme, the sector was allowed to configure, in a bottom-up manner, interorganisational networks within a chosen geographical area. In a first stage we analyze the plans and underlying meaning given by the projects to this government programme, as part of a longer term prospective programme evaluation of interorganisational networks in mental health care.

**Methods:**

Literature on inter-organizational networks, content analysis of project-proposals and in-depth interviews.

**Results and conclusions:**

The proposed network configurations differ widely in type, size, heterogeneity and complexity. Many projects build their network model strongly upon previous informal collaborations and reproduce informal partnerships within a new government framework.

**Discussion:**

A policy approach offering a framework with which the providers can construct their proper intra-organisational network logic, leads to a wide variety of interorganisational network configurations and collaboration models. These networks can be sufficiently adapted to the local contexts, but further research will be needed to assess their effectiveness and efficiency for providing integrated care.

